# Management of Lumbar Abscess Secondary to Colocutaneous Fistula From Complicated Diverticular Disease Using Negative Pressure Therapy

**DOI:** 10.7759/cureus.73871

**Published:** 2024-11-17

**Authors:** Saul Xicohtencatl, Diomedes Durango, Roberto Elías Damacio, Daniela Cabrera

**Affiliations:** 1 General Surgery, Instituto de Seguridad y Servicios Sociales de los Trabajadores al Servicio de los Poderes del Estado de Puebla, Puebla, MEX; 2 Radiology, Instituto de Seguridad y Servicios Sociales de los Trabajadores al Servicio de los Poderes del Estado de Puebla, Puebla, MEX

**Keywords:** colocutaneous fistula, diverticulitis, fistula, vac, veraflo

## Abstract

Diverticular disease is a common gastrointestinal condition with rising prevalence. Complications, such as fistulas, are rare but significant, often requiring innovative treatment strategies. This case report examines the use of negative pressure wound therapy (NPWT) with instillation (VERAFLO®, KCI, an Acelity Company, San Antonio, Texas) and antiseptic solution (VASHE®, Urgo Medical North America LLC, Fort Worth, Texas) in treating a colocutaneous fistula secondary to complicated diverticular disease. A 43-year-old male presented with lumbar pain and erythema. Computed tomography (CT) scans revealed a left-sided collection with gas, suggesting a colocutaneous fistula. Initial management included drainage, antibiotics, and NPWT. Follow-up involved transrectal contrast CT to assess the fistula tract and subsequent colonoscopy to confirm resolution. NPWT with VERAFLO® was applied for 20 days, followed by wound closure. Two-month follow-up showed no evidence of fistula on colonoscopy, confirming successful treatment. NPWT can be an effective alternative for treating colocutaneous fistulas in diverticular disease, promoting wound healing and reducing infection. Further research is warranted to explore its broader applications in gastrointestinal fistulas.

## Introduction

Diverticular disease of the colon is a common gastrointestinal (GI) condition that has increased in prevalence over recent decades. This condition can range from three clinical varieties that are diverticulosis, which presents asymptomatically and incidentally by radiological or endoscopic studies; on the other hand, the clinical variety in terms of its presentation associated with symptoms varies according to the severity of the clinical picture with symptoms of pain and without complications named symptomatic uncomplicated diverticular disease (SUDD), as well as complications with diverticulum perforation with clinical data of abdominal pain, fever, signs of peritonitis, abscesses, fistulas, and bleeding. The diagnosis requires a combined assessment of clinical signs, biomarkers, and cross-sectional imaging, particularly the last one in cases of acute diverticulitis, using methods such as ultrasonography, computed tomography (CT), and magnetic resonance imaging (MRI). The colonoscopy is usually contraindicated in the first six to eight weeks near the acute diverticulitis [1]. Risk factors include a low-fiber diet, consumption of red meat, advanced age, gender, smoking, and the use of nonsteroidal anti-inflammatory drugs (NSAIDs) [2]. Treatment usually includes dietary fiber, antibiotics, anti-inflammatory drugs, and probiotics [1].

Fistulas are a rare and serious manifestation, representing a small percentage of complicated diverticulitis cases, and usually occur as a postoperative complication or following abscess drainage [3]. Management requires the application of a care protocol named the acronym “SNAP,” which includes management of sepsis control, nutritional support, delineation of anatomy, and a plan for definitive surgical repair. The first step requires early goal-directed fluid resuscitation and electrolyte replacement, application of broad-spectrum antibiotics and source control with percutaneous or open fistula with wound care, and effluent control with a wet-to-dry dressing or simply a dry gauze or in high-output fistulas using collection devices, such as ostomy appliances, wound managers, pouching systems that can be connected to wall suction a negative pressure wound therapy (NPWT). Nutritional support with enteral and/or parenteral nutrition to respond in front of three sources of malnutrition: inadequate calorie intake, catabolism related to ongoing sepsis, and ongoing losses from the GI tract. To define the fistula anatomy, it is necessary that imaging studies be performed at least seven to 10 days after stabilization with conservative management and identify any postoperative, neoplastic, infectious, or inflammatory condition to contribute to the enterocutaneous fistula (ECF). Definitive operative intervention gives the best chance of cure from enterocutaneous fistula and re-establishing bowel continuity whenever possible if it does not close spontaneously; the optimal surgical time recommended is six to 12 months after ECF onset with open or endoscopic procedures [4]. Although the use of NPWT, such as vacuum-assisted closure (VAC) for intestinal fistulas, was historically discouraged and not specially approved for ECF, recent studies have demonstrated its effectiveness in some cases, controlling fistula output, wound care, and spontaneous closure of the fistula [5].

## Case presentation

A 43-year-old man presented at a hospital in Puebla, Mexico, on October 21, 2023, with lumbar pain, swelling, and erythema of 10 days' duration. These symptoms had previously been treated at another facility as a muscle contracture. Laboratory tests showed leukocytosis of 28,000/mm^3^ and neutrophilia of 88%.

**Figure 1:**
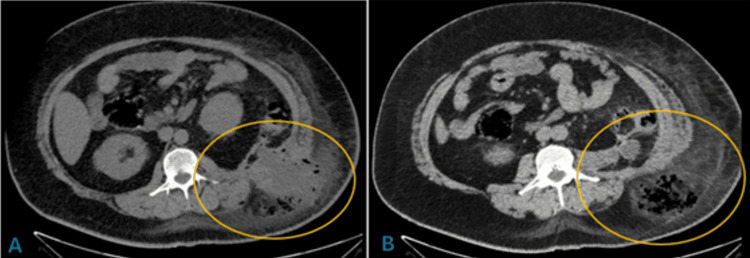
Non-contrast axial abdominal CT scan. (A) A heterogeneous collection at the left flank associated with gas and surrounding fat stranding communicating into the abdominal cavity, involving the colon. (B) Heterogeneous collection is associated with gas and surrounding fat stranding in soft tissues.

A non-contrast abdominal CT scan with axial, coronal, and sagittal slices revealed a hypodense area (23 HU) in the left flank, extending into the deep fascia of the ipsilateral renal fossa, with the presence of gas (-236 HU) and an apparent transition zone into the retroperitoneum (Figures 1a, 1b).

**Figure 2:**
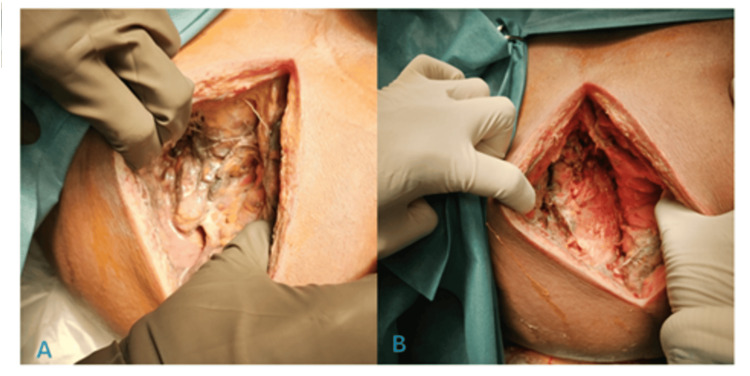
Transoperative findings. (A) and (B) Show a bloody area in the left lumbar region following the drainage of a mixed content collection, including fecal and purulent material.

The collection was drained with 500 ml of output with fecal matter, and the surgical site was cleaned, incorporating a presumptive diagnosis of acute complicated diverticulitis with retroperitoneal lumbar abscess based on the characteristics of the secretion (Figures 2a, 2b).

**Figure 3:**
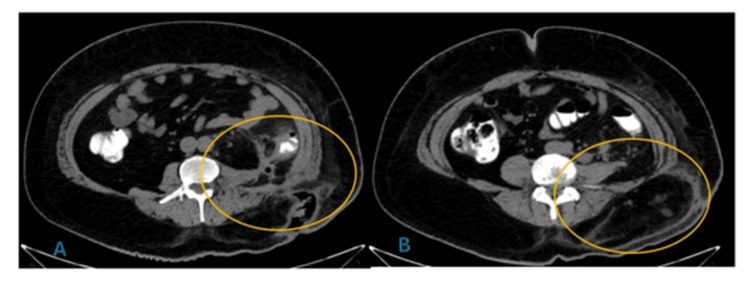
Axial abdominal CT scan with water-soluble contrast administered rectally. (A) Yellow markings indicating a fibrotic band retracting the descending colon and left psoas muscle extending into the fistulous tract, with no extravasation of contrast medium into the abdominal cavity. (B) Saccular image at the junction of the descending colon and sigmoid colon protruding beyond the wall.

Initial medical management for the fistula was performed according to the Evenson and Fischer phases. After draining the source, in the stabilization phase, treatment included antibiotics, oral dietary restriction, total parenteral nutrition, and local control of the fistula drainage with negative pressure therapy using the VERAFLO® (KCI, an Acelity Company, San Antonio, Texas) instillation mode and VASHE® antiseptic solution (Urgo Medical North America LLC, Fort Worth, Texas). Subsequently, in the investigation phase, a contrast-enhanced abdominal CT scan with transrectal contrast was performed, identifying at least two saccular lesions in the descending colon protruding beyond the wall and extending into the fistulous tract. In the left lumbar region at the level of L2, a fistulous tract was identified connecting the peritoneal cavity with the skin, presenting with air and fluid and surrounding fat stranding (Figures 3a, 3b).

**Figure 4:**
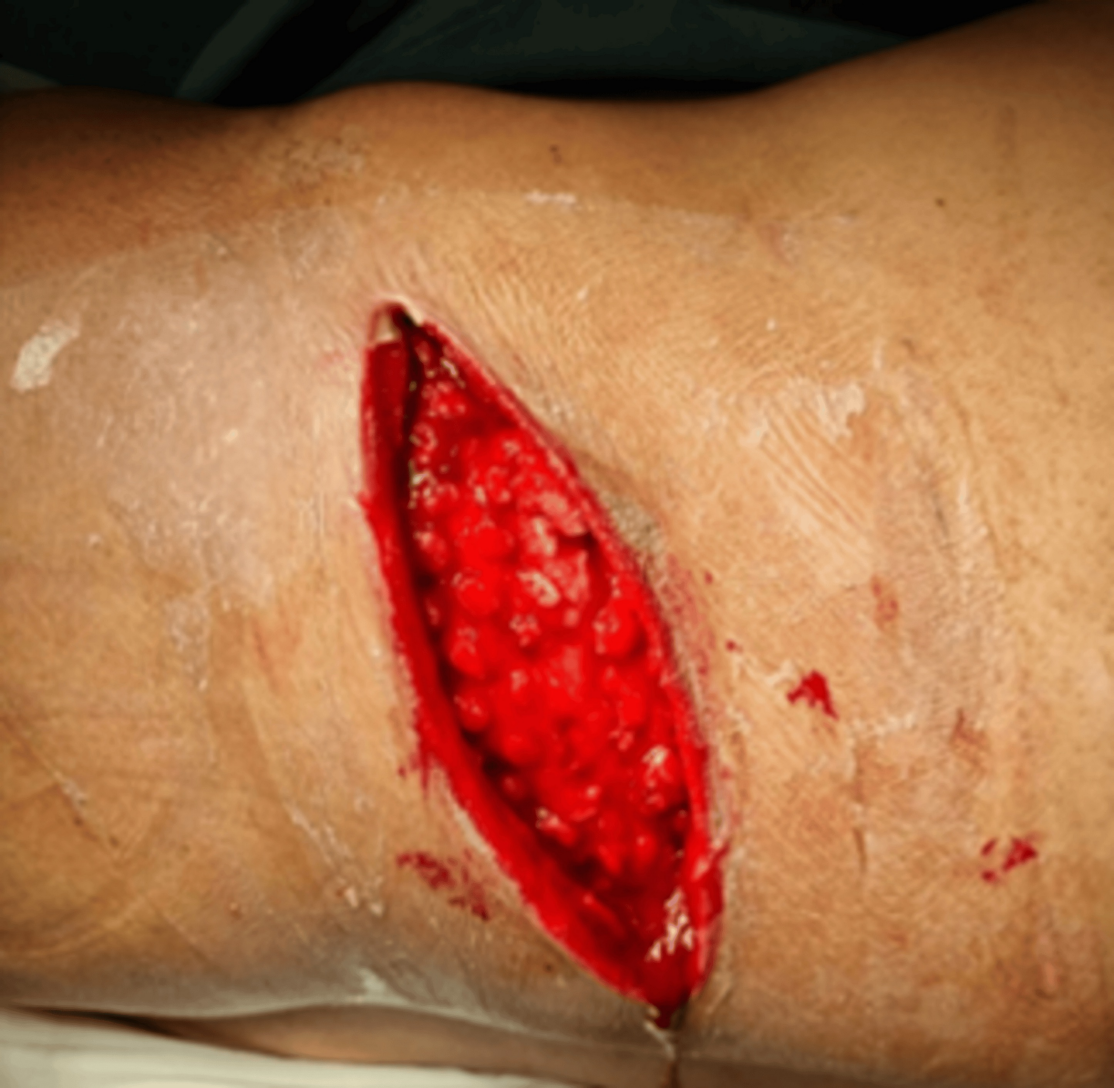
Postoperative findings. Bloody area in the left lumbar region, clean after the removal of VAC therapy. VAC: vacuum-assisted closure.

**Figure 5:**
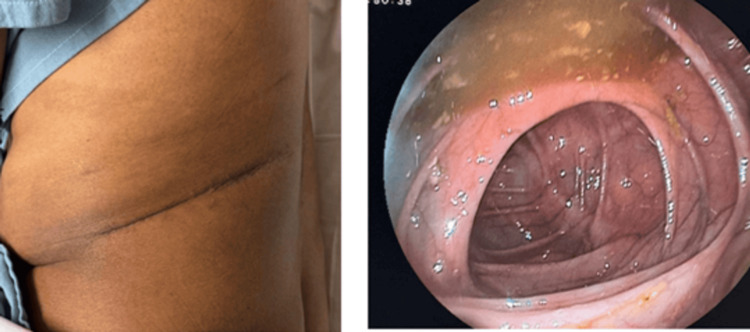
Late evaluation after two months. Left: Clinical findings with a closed wound with no evidence of fistula at follow-up two months after VAC system removal. Right: Colonoscopy showing no evidence of fistula. VAC: vacuum-assisted closure.

The decision was made to discharge the patient with external follow-up via outpatient consultation, with an apparent resolution of the clinical condition. Two months later, a colonoscopy confirmed an intact colon, resolution of the condition, and closure of the colocutaneous fistula (Figures 4, 5).

## Discussion

Diverticular disease of the colon is one of the most frequently diagnosed GI conditions. Symptomatic diverticular disease presents a broad spectrum of severity, ranging from abdominal pain to perforation, peritonitis, and sepsis. Complicated diverticular disease is characterized by free perforation, abscess formation, and complications such as fistula, stenosis, and obstruction [2].

The prevalence of diverticular disease has increased over recent decades. According to the latest NIS report, hospitalization rates for diverticulitis have risen from 74 per 100,000 in 2000 to 96 per 100,000 in 2008. More recent data indicate that in 2014, 1.92 million patients were diagnosed with diverticular disease in an outpatient setting [2].

Various terms have been used to describe the condition, leading to some confusion. The European Society of Coloproctology terms include diverticulosis, which denotes the presence of diverticula, and diverticulitis, which describes the presence of symptoms. Diverticulitis refers to inflammation of the colonic wall and often adjacent tissues. It can occur acutely or chronically and may be associated with complications [6].

When diverticulitis is not fully resolved, it can progress to a chronic form, manifesting as stenosis that may lead to obstruction or a fistula, often with the urinary tract [6]. Several risk factors are associated with diverticular disease, including low dietary fiber intake, a Western diet (high in red meat, refined grains, and fats), genetic predisposition (with up to 40-50% heritability risk), age (incidence increases to 80% after age 80), gender (more common in men with a 3:1 ratio), smoking, and NSAID use [2].

The traditional pathogenesis concept involves the development of diverticula associated with chronic constipation, leading to increased intraluminal pressure and protrusion of mucosa and submucosa through the muscular layer at the site of blood vessel entry (vasa recta), predominantly in the sigmoid colon and left colon [7].

Historically, 10-25% of patients with diverticulosis develop diverticulitis. Chronic forms of diverticulitis are characterized by the formation of fibrotic stenotic tissue and fistulas, typically to the bladder, vagina, and occasionally to the small intestine and skin [7].

Diverticular fistula, defined as an abnormal connection between the colon and the skin or another organ, is a rare manifestation of diverticular disease, representing 3.12% of all admissions for diverticular disease. It is considered an indication for surgery and accounts for 5-9% of surgeries for diverticulitis [8].

Complicated diverticulitis requires management with percutaneous or open abscess drainage, depending on the site of the liquid collection. In 6.3% of cases, a colocutaneous fistula occurs post-drainage and requires a two-stage operation [9].

Colocutaneous fistulas typically present in the left flank and hip [10]. In the context of enterocutaneous fistulas, classification varies by output and etiology. Approximately 75-85% are post-surgical, while 15-25% are spontaneous, arising from inflammatory bowel disease, malignancy, appendicitis, and diverticulitis [4].

The rates of spontaneous closure without surgical intervention in the era of advanced wound care and parenteral nutrition vary considerably, ranging from 20% to 30% [4]. With advanced wound care, spontaneous closure of the fistula occurs in 90% of cases within the first month [11]. Negative pressure therapy and other negative pressure treatments have reported cases of fistula closure in the second or third month [4].

Vacuum-assisted closure (VAC) is a widely known method for closing traumatic and chronic wounds, first used in the 1990s for chronic wound treatment. Its complex mechanism reduces edema and wound exudate while promoting granulation. Another known mechanism is the reduction of bacterial content. Until recently, VAC was contraindicated for managing intestinal fistulas, as it was believed to prolong healing time and potentially cause damage to internal organs. Currently, several studies have demonstrated the efficacy of VAC in certain types of fistulas, mostly simple fistulas with a single opening to the skin and post-surgical fistulas [5].

VAC is a type of negative pressure wound therapy not specifically approved for enterocutaneous fistulas, but with increasing application and controversial results, both positive and negative. In a series of 92 patients with high-output fistulas managed with VAC, spontaneous closure occurred in 46% and output control in 98%. Medeiros et al. reported 74 patients with enterocutaneous fistulas, with 92% achieving spontaneous closure within 15 days. Two case series reported fistulas associated with VAC therapy [4].

However, there is no clear evidence that VAC therapy improves fistula closure, and in some cases, it may be harmful [4]. Although several studies have reported fistula closure with VAC therapy, its main advantages include reducing fistula output and promoting the healing of skin lesions while keeping the skin dry [12].

In our present case, a systematic approach to the fistula was used, opting for vacuum-assisted closure therapy, considering its advantages in controlling local infection, promoting granulation, and indirectly using pressure for fistula tract closure.

## Conclusions

Diverticular disease is one of the most common GI conditions, with increasing prevalence and incidence over the past two decades. Consequently, it is important to consider its rare complications. In cases of complicated diverticular disease, unusual presentations such as fistula formation can occur, as demonstrated in this case.

While the standard treatment recommended by the American Society of Colon and Rectal Surgeons and the European Society of Coloproctology is surgical, there are alternative treatments reported in the literature, with an approach to control sepsis with drainage, lavage with antiseptic solution, assisting spontaneous closure in less time and interventions with NWPT. Although this therapy is not specifically approved for this condition and its use is controversial compared to endoscopic therapies where a delimitation of the anatomy of the fistula is required with identification of the primary orifice of the same, having options such as (clips, plugs, sealants, and stents).

In this case, we used a systematic approach to treat a diverticular fistula with NPWT in the first step of the SNAP protocol, achieving favorable outcomes and subsequently confirming fistula closure without a second surgery. The use of VAC therapy for managing fistulas is an underexplored area with potential for future expansion.
